# Comparative Study on Strengths of Ready-to-Assemble and Eccentric Furniture Joint

**DOI:** 10.3390/ma18092114

**Published:** 2025-05-04

**Authors:** Nikola Janíková, Adam Kořený, Milan Gaff, Josef Hlavatý

**Affiliations:** Design and Furniture, Faculty of Forestry and Wood Technology, Mendel University in Brno, 613 00 Brno, Czech Republicmilan.gaff@mendelu.cz (M.G.);

**Keywords:** innovative furniture joints, eccentric CAM joint, bending moment, corner joint, modern connecting elements

## Abstract

This study compared two groups of furniture joints, i.e., a so-called ready-to-assemble (RTA) plastic biscuit joint from Lamello©, while the second group consists of four types of eccentric joints with beech dowels. L-shaped specimens were prepared with the help of the selected joints and a three-layer particleboard with dimensions of 150 × 150 × 400 mm. These L-shaped specimens were tested for bending moment capacity under compression and under tension. Cam joints with wooden dowels can withstand high stress. If Lamello© Bisco P-15 joints are added to the plastic Clamex P-14 joint, this joint will achieve 13% higher values for bending moment capacity under compression and 22% under tension. During testing, the worst result was achieved by the Tenso P-14 joint. The best values achieved during the testing of bending moment capacity under compression and under tension were for an eccentric joint with the use of a metal-capped bolt and Euro screw. This joint achieved 147% higher values for bending moment capacity under compression than a standard eccentric joint with a euro screw bolt and 213% higher values for bending moment capacity under compression than the Lamello© and Clamex P-14 joints. This study aimed to determine how the joints differ, how they behave during testing, and what deformations occur.

## 1. Introduction

Furniture manufacturers have been striving to find the most efficient methods for connecting furniture parts since time immemorial. This field continues to evolve, with ongoing efforts to identify optimal solutions that meet the demands for faster production, a reduced number of manufacturing operations, and cost optimization while also addressing aesthetic and functional requirements, such as joint visibility and disassembly options. However, ensuring sufficient joint strength remains crucial (Janikova et al., 2025) [1].

Recent innovations include new types of joints manufactured from combinations of plastic and metal alloys. Karaman (2020) [2] investigated the performance of L-shaped joints using Lamello© plastic connectors, specifically examining the Tenso P-14 joint and the Clamex P-14 joint, with the latter integrating a zinc component to strengthen the connection. The tested specimens measured 150 × 150 × 150 mm, with different adhesives applied to each sample. In a subsequent study, Karaman (2021) [3] focused on the positioning of RTA plastic joints, studying the effect of the distance of the Clamex P-14 connector from the edge. Another part of the study examined the addition of wooden dowels, made from various wood types (oriental beech, oak, Scotch pine), to the Clamex P-14 joints.

Silvana et al. (2019) [4] tested L-shaped specimens subjected to angular plane bending. Their 106 × 106 × 100 mm specimens included various joints, such as the Minifix eccentric CAM joint (with 24 mm and 34 mm screws), the Clamex P-14 demountable plastic connector, and the fully concealed metal Invis joint, combining a metric thread and a confirmat screw. Similarly, Sydor and Pohl (2019) [5] compared plastic Clamex P-10 RTA connectors with eccentric CAM connectors.

Karaman et al. (2020) [6] explored novel joint types produced using 3D printing, comparing biscuits made from polylactic acid (PLA) and acrylonitrile butadiene styrene (ABS) to traditional beech wood biscuits. Their results indicated comparable strength levels between the 3D-printed and wooden biscuits. Atar et al. (2009) [7] and Tankut (2004) [8] also studied wooden biscuits in L-shaped specimens constructed from various sheet materials, achieving better results with melamine-coated fiberboard and miter joints. Alar et al. (2022) [9] investigated corner joints with dowels, CAM joints, and traditional half-blind dovetail joints, evaluating different adhesive types. Zhang et al. (2024) [10] studied CAM joints terminated with metal or plastic dowels and compared their performance to that of conventional bolts, screws, and wooden dowels. Kureli and Altinok (2011) [11] and Šimek and Konas (2009) [12] both focused on the bending behavior of L-shaped specimens with eccentric CAM joints under angular plane loading, with the latter also including numerical simulations.

Janikova et al. (2024) [13] compared eccentric CAM joints with plastic connectors, while Krzyzaniak and Smardzewski (2020) [14] evaluated fully concealed plastic joints versus eccentric CAM joints with steel screws and plastic swivels. Smardzewski et al. (2014) [15] conducted similar tests using confirmation screws and eccentric CAM joints in specimens of varying sizes (150 mm and 500 mm). Guo et al. (2019) [16] and Kucuktuvek et al. (2017) [17] both examined eccentric CAM joints featuring M4 metric threads, testing them in particleboard and poppy husk-based particleboards.

Hu et al. (2024) [18,19] investigated eccentric CAM joints combined with nylon dowels, analyzing both bending performance and pull-out strength in particleboard, plywood, and block board specimens with varying surface layer thicknesses. Yerlikaya (2012) [20] compared joints with and without fiberglass edge reinforcement. Skorupińska et al. (2022) [21] studied the strength of nuts in different materials, including softwood, OSB, plywood, and particleboard.

Jivkov (2002) [22] examined the effects of ABS edge banding in joints connected with CAMs, confirmat screws, and dowels. Jivkov et al. (2021) [23] tested lightweight panels made from recycled cardboard and beech veneer connected with plastic connectors, confirmat screws, CAMs, and wooden dowels.

Karaman (2024) [24] studied H-shaped specimens connected by eccentric CAM joints and screws, combined with beech or oak dowels.

Reliability and cyclic testing of furniture joints were the focus of Klos and Langová (2023) [25], who studied dowels, confirmat screws, and eccentric CAM joints. Máchová et al. (2019) [26] and Klos et al. (2018) [27] further tested L-shaped specimens under angular bending, comparing various materials and joints.

Kasal et al. (2008) [28] and Kasal et al. (2010) [29] examined permanent screw connections in particleboard and fiberboard materials, analyzing variations based on screw size and number.

Bas et al. (2024) [30] evaluated the Festool Domino dowel and demountable Domino connectors, comparing their performance with traditional glued joints. Smietańska and Mielczarek (2022) [31] also studied the application of Domino connectors in L-shaped specimens made of particleboard and MDF.

Kasal et al. (2020, 2023) [32,33] and Kuskun et al. (2020, 2023) [34,35] explored the use of auxetic plastic dowels as innovative joint solutions, focusing on optimizing dowel shapes to improve mechanical performance. Smardzewski et al. (2016) [36] investigated corner joints with innovative plastic push-lock connectors, while Branowski et al. (2020) [37] developed new eccentric CAM structures. Petrova and Jivkov (2024) [38] tested L-shaped specimens connected with external plastic locking elements, and Sydor et al. (2021) [39] introduced T-shaped corner joints made of plastic–metal combinations.

Previous research has predominantly studied lamella-type and eccentric joints separately, limiting direct comparison between these two categories.

This study aims to address this gap by directly comparing plastic lamellar joints and various eccentric joints under identical testing conditions. Using the same materials and testing methodology, this research enables an objective comparison of the performance characteristics of each joint type.

Lamello© fasteners (Clamex and Tenso) are particularly valued for their quick assembly and disassembly capabilities and the ability to reposition connected parts. However, their unit cost is significantly higher than that of eccentric joints. This raises user expectations regarding joint strength, especially considering the greater production complexity associated with machining grooves for Lamello© systems.

Therefore, this research compares Lamello© joints with traditional eccentric joints to evaluate their relative advantages and disadvantages.

The primary objective of this study was to compare popular Lamello© joints with eccentric joints using identical materials and testing protocols to ensure fair comparison. Another aim was to compare different types of lamella joints and to assess whether, and to what extent, the addition of a middle plastic lamella improves the strength of the Clamex P-14 joint.

## 2. Materials and Methods

### 2.1. Material

Seven types of joints were selected for the research and divided into two groups: RTA joints (specimens A, B and C) and eccentric CAM joints (specimens D, E, F and G) (Table 1).

The RTA joint group is represented by plastic biscuit joints (Lamello© Clamex P-14 and Tenso P-14), which have a groove for joint installation. These joints are produced in Switzerland. For the group of eccentric CAM joints (produced by a company in Germany), the only difference between specimens was the type of bolts.

Twenty replicates of each type of L-specimen were made with dimensions of 150 × 150 × 400 mm. The face and rear members were joined at a 90° angle. The base material was a three-layer particleboard (producer: DDL Lukapol from Slovakia; basic with lamination; 18 mm thickness). For the production of particleboards, chips of coniferous wood were used, which were bonded with urea–formaldehyde glue. Lukapold boards meet the requirements of emission class E1, meaning they have a low level of formaldehyde emissions. They achieve values of <0.1 ppm according to EN 16516 and <0.05 ppm according to EN 717-1, significantly below the maximum limits for class E1. They are classified as type P2 according to the EN 312 standard—suitable for general use in dry conditions, particularly in furniture manufacturing and interior design. Each specimen has a 0.5 mm thick ABS edge. This edge was bonded with polyurethane adhesive (Hranitherm 860 PUR) from Hranipex, a company based in the Czech Republic. A total of 140 L-shaped specimens were made.

### 2.2. Sample Preparation

**Figure 1:**
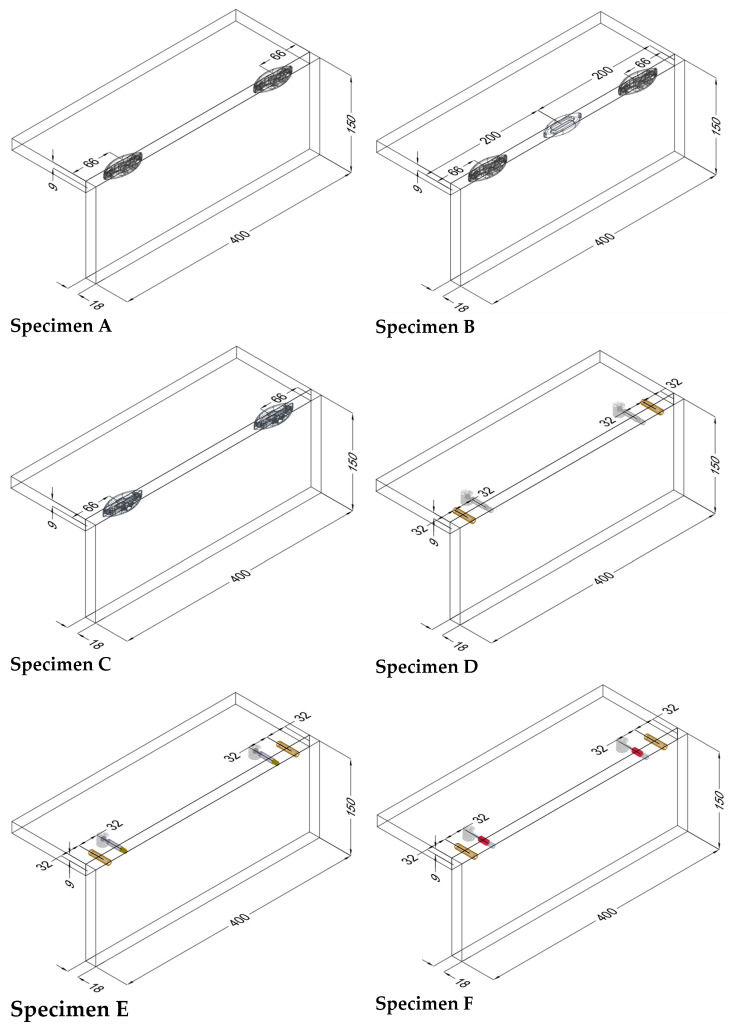

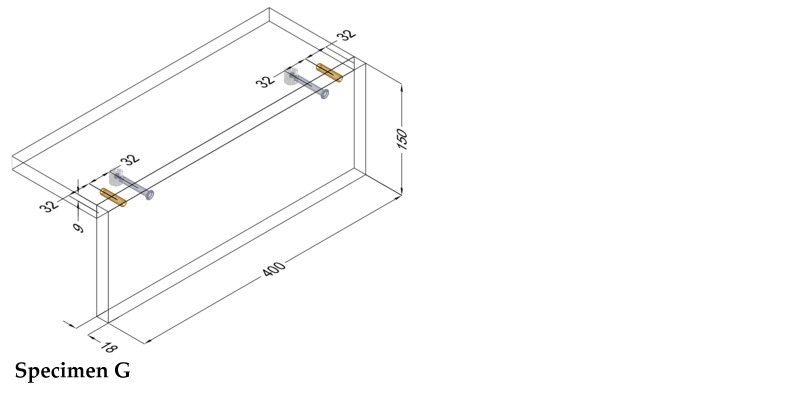
Schematic of the L-type corner joint for specimen types A, B, C, D, E, F and G. Measurements in millimeters.

Figure 1 shows details like the dimensions and positions of the joints. Table 1 summarizes the details of the components used, including their numbers, the name of the producer, and their serial numbers.

Table 1 lists selected RTA joints (group A–C) and eccentric CAM joints (group D–G), showing photographs of the components used in the joint, their amounts, the type’s size, and the presence of milling, if applicable.

**Table 1:** Selected types of joints and components.

Specimen’s Type	Parts Used in Joint Connection	Number of Fittings in the Joint Connection
A	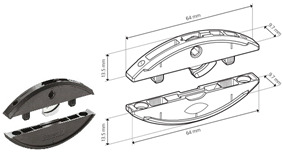	• 2 pcs Clamex P-14 (Lamello©—145334)
B	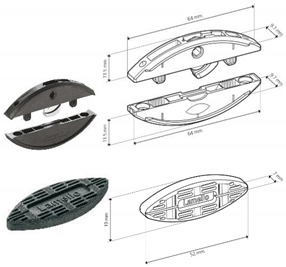	• 2 pcs Clamex P-14 (Lamello©—145334) • 1 pcs Bisco-P15 (Lamello©—145304)
C	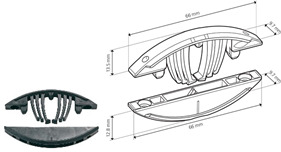	• 2 pcs Tenso P-14 (Lamello©—145415)
D	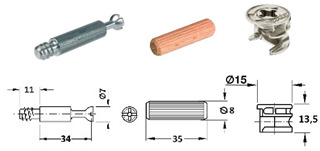	• 2 pcs steel bolt S100 (262.28.026) • 2 pcs Minifix^®^ 15 (262.26.034) • 2 pcs beach dowel (262.82.235)
E	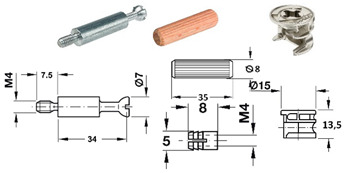	• 2 pcs steel bolts S100 M4 (262.28.937) • 2 pcs Minifix^®^ 15 (262.26.034) • 2 pcs insert nut M4 (051.45.004) • 2 pcs beach dowel (262.82.235)
F	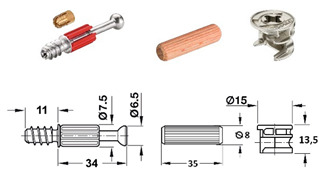	• 2 pcs steel bolt S200 (262.28.670) • 2 pcs Minifix^®^ 15 (262.26.034) • 2 pcs beach dowel (262.82.235)
G	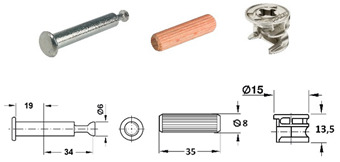	• 2 pcs capped bolt (262.28.765) • 2 pcs Minifix^®^ 15 (262.26.034) • 2 pcs beach dowel (262.82.235)

**Figure 2:**
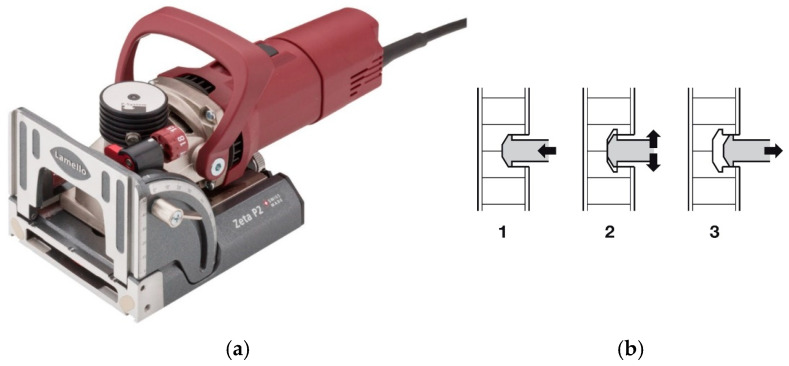
(**a**) Lamello© groove milling machine Zeta P2 1050 W (with an illustration of the milling groove, (**b**) 1–3 individual steps of groove milling) (Company Lamello AG from Switzerland, city Bubendorf).

The specimen parts were produced with a Holzma HPP 250 machine (produce by company Homag from Germany, city Schopfloch) and an OTT Tornado+ (produced by company Paul OTT GmbH from Austria, city Lambach) edge bander. The structural work was performed manually. The original Lamello© groove milling machine Zeta P2 1050 W (Figure 2) with a disk and profile cutter, which can mill a groove in three steps while also cutting the necessary profile for inserting the joint into the material (Figure 2), was used for the production of specimen types A, B and C. The joint is characterized by a groove shape manufactured using the procedure shown in Figure 2. The manufacturer’s original accessories ensure the accuracy of the machining. If accessories other than the original are used, an appropriate technological process should be ensured.

**Figure 3:**
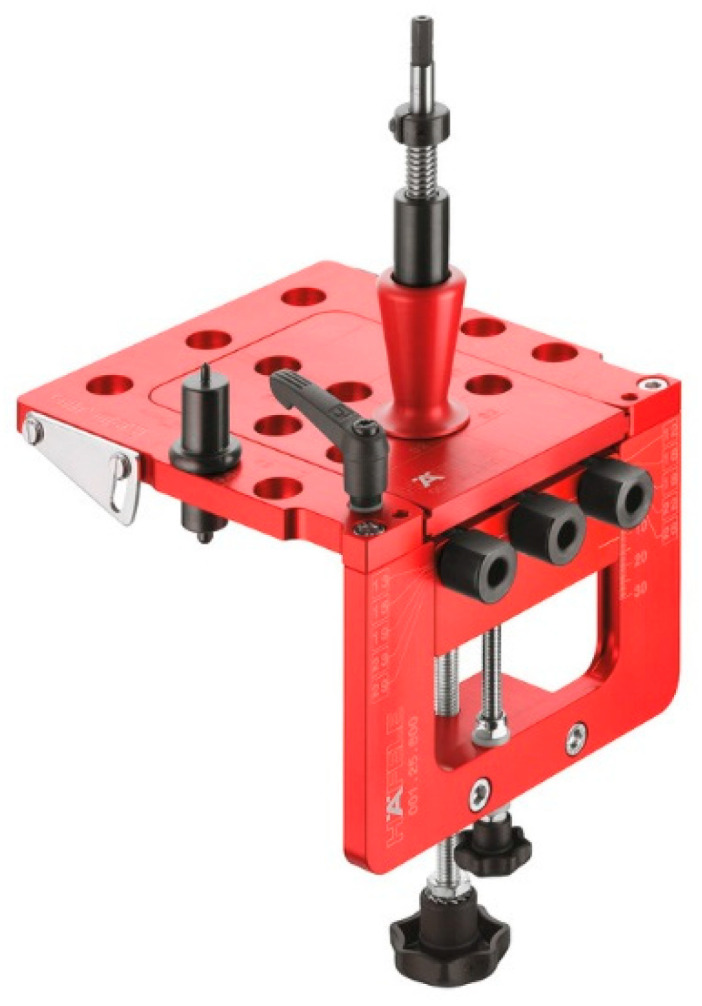
Universal drilling template with interchangeable depth stops (Red Jig 001.25.890).

A universal drilling template from a German company, Red Jig with order number 001.25.890 (Figure 3), with interchangeable depth stops was used to produce specimens D, E, F and G. Side stops and the measuring system on the template combined with the depth stops on the drills guaranteed the precise drilling of each sample. No adhesive was used for specimen type C (Lamello© Tenso P-14), although the manufacturer recommends it. Using adhesive would make the demountable RTA joint a permanent joint. The joint element can only hold the two parts until the adhesive has hardened. The connecting element allows the two parts to move relative to each other, but using adhesive would eliminate the joint type’s advantage in disassembly. There was also no adhesive used on the wooden dowels in joints D, E, F and G.

In common practice, manufacturers appreciate the possibility of quick assembly and the option of disassembly available with the C type joint (Lamello© Tenso P-14), and they do not use the adhesive specified by the manufacturer. The adhesive would only allow the user to move the pieces together and fix them until the adhesive in the joint has cured. However, given the price of the connector, we cannot expect that manufacturers would use this connector specifically for this purpose.

### 2.3. Method of Testing

**Figure 4:**
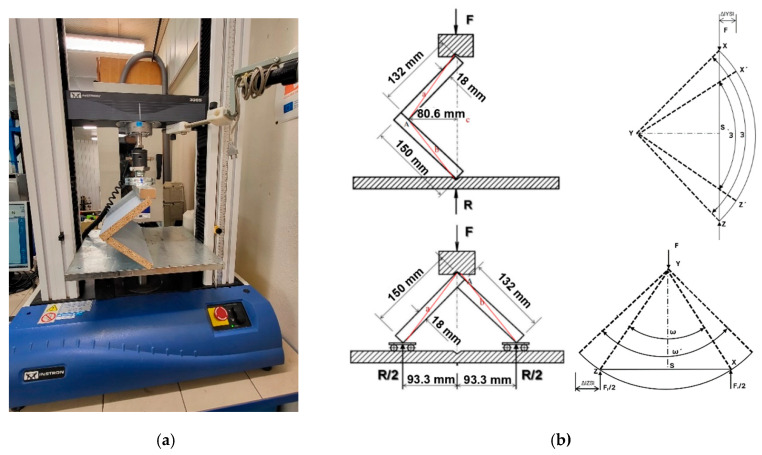
Universal testing machine with (**a**) test set-up for joints and (**b**) schematic presentation of the compression and tension tests.

The universal testing machine Instron 3365 (produced by Instron Corporation from USA, city Norwood in Massachusetts) was used to test L-shaped specimens with the addition of sliding plates (Figure 4). The testing machine applied force to the specimens at a constant 8 mm/min feed rate. The magnitude of the applied force and the displacements of the parts of the tested specimens were automatically recorded and subsequently analyzed (Figure 4).

STATISTICA 13 was used to analyze the results with a one-way analysis of variance (ANOVA). The results were tested at a significant level of 95%. Interaction within each sample was tested with the Tukey HSD test, which determined whether certain groups of specimens were similar.

## 3. Results and Discussion

### 3.1. Results of Mechanical Testing

**Figure 5:**
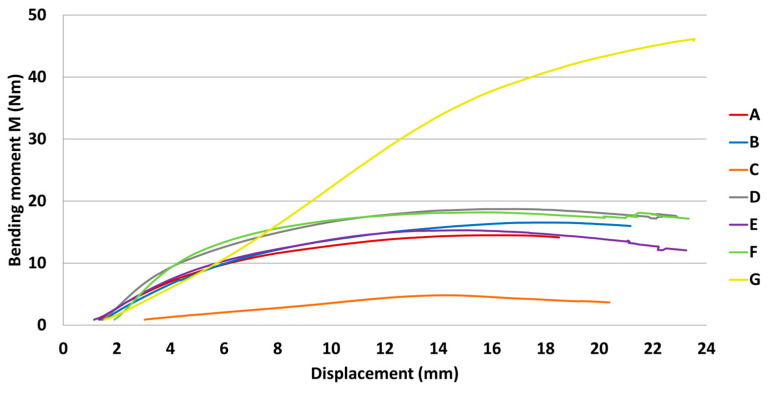
Graph of bending moment capacity under compression.

**Figure 6:**
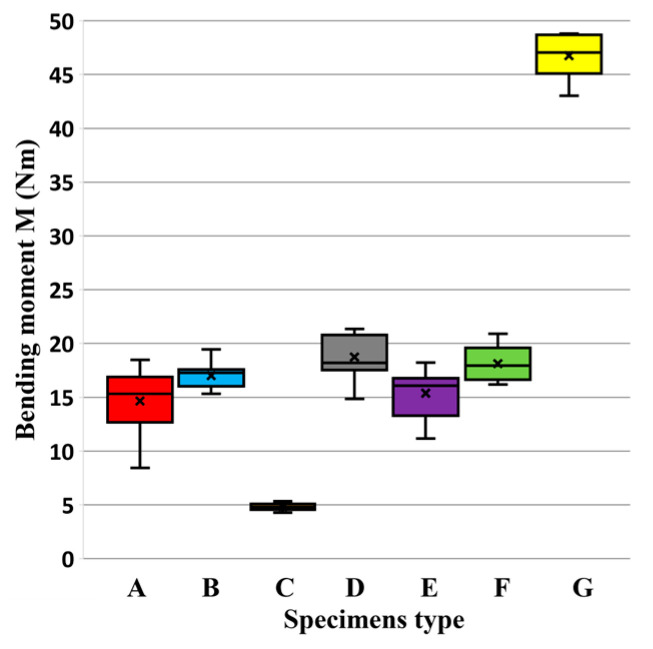
Box plot of bending moment capacity under compression.

**Figure 7:**
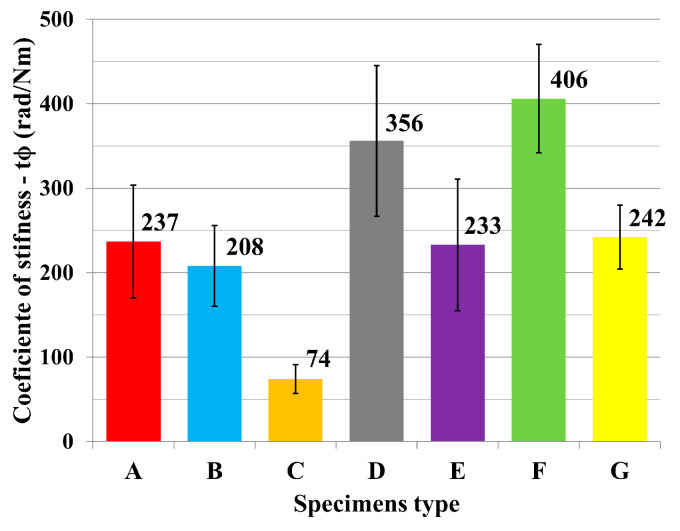
Stiffness of tested joints under compression.

The results of mechanical testing for bending in an angular plane are presented in the tables and graphs. The result of bending in an angular plane was compression (Table 2) and tension (Table 3), where the average, measured in Nm, and the stiffness, measured in Nm/rad, are listed for each tested type. A regression analysis of bending in an angular plane—compressive stress—is shown in Figure 5, where we can see the course of the test and displacement. This test is also shown in a box plot (Figure 6). The stiffness results are shown in Figure 7.

Research hypothesis H0 was established; there is an insignificant difference between the mean values. Hypothesis H0 assumes that the measured means between groups are the same or differ only by chance. A significance test was performed with a significance level of α = 0.05, which demonstrated differences between the individual groups of tested specimens. Hypothesis H0 was rejected because the tested criterion was greater than the critical value (444.23 > 2.25). The type of joint has a statistically significant effect on the measured Mmax. The mean values of the core sets from which the analyzed selections were taken differ (Table 4). The results of mechanical testing have shown differences between different types of joints.

According to the one-way ANOVA and pairwise comparison conducted with the Tukey HSD test, a significant difference of α = 0.05 and a level of confidence of 95% were found between joints (Table 4).

A two-way analysis of variance (ANOVA) (Table 5) was conducted to examine the effects of the factors Sample and Columns on the measured outcome. The analysis revealed that both main effects and their interaction were statistically significant. The Sample factor had a highly significant effect on the dependent variable (F(6,126) = 352.97, *p* < 0.001), indicating that different samples influenced the results to a significant extent. The Columns factor also showed a strong effect (F(1,126) = 145.57, *p* < 0.001), suggesting that this grouping variable contributed substantially to the variation observed. Furthermore, a significant interaction effect between Sample and Columns was detected (F(6,126) = 96.94, *p* < 0.001), indicating that the influence of one factor depends on the level of the other.

The joints were divided into four groups in the Tukey HSD test (Table 6), with three joints in two groups and only one in the remaining two. The first group consists of plastic biscuit joints A and B, between which there was a 16% difference during testing. This group also includes joint E, an eccentric joint with an M4 bolt and an unglued beech dowel. The second group consists of eccentric joints D and F, between which the tests showed a difference of 3.3%, as well as joint B, a plastic RTA joint with a plastic biscuit in the middle.

**Figure 8:**
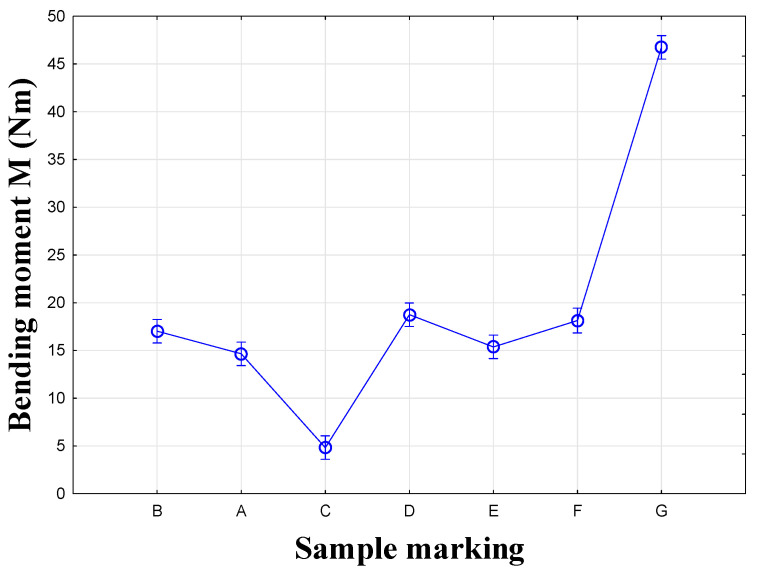
Graph of results for the compression test.

The third and fourth groups each contain one sample, C and G, which did not resemble any other joint in terms of their test results. The average values of the maximum moment (Mmax) for each type of connection are given in Figure 8. The lines represent the standard deviation. The results show that two basic groups of specimens were mixed together: RTA joints (specimens A, B and C) and eccentric joints (specimens D, E, F and G). Joint B (Lamello© Clamex P-14 + Bisco P-15) connected these groups.

**Figure 9:**
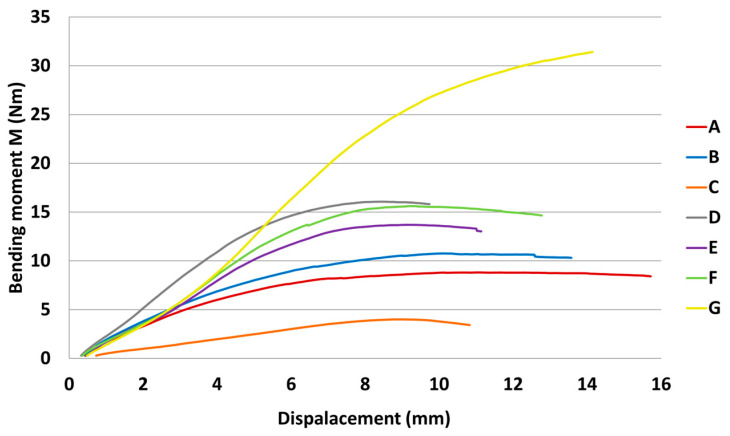
Graph of bending moment capacity under tension.

**Figure 10:**
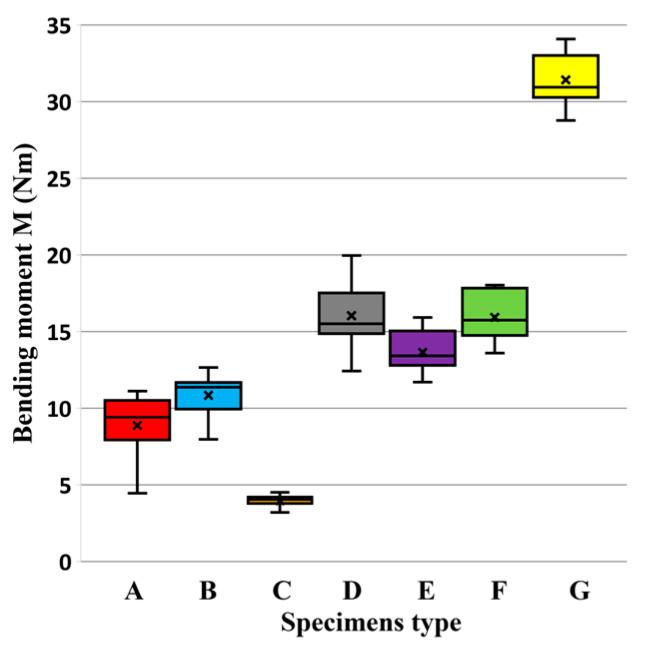
Box plot of bending moment capacity under tension.

**Figure 11:**
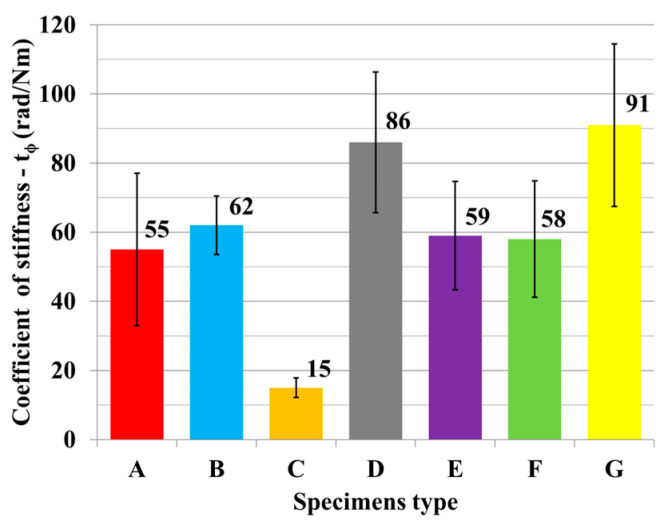
Stiffness of tested joints under tension.

The results of bending in an angular plane—tensile stress—are shown in Table 4, where the average measured values and the stiffness Nm/rad are listed for each tested type. The regression analysis of bending in an angular plane—compressive stress—is shown in Figure 9, where we can see the course of the test and displacement. This test is also depicted in a box plot (Figure 10). The stiffness results are shown in Figure 11.

Research hypothesis H0 was established; there is an insignificant difference between the mean values. Hypothesis H0 assumes that the measured means between groups are the same or differ only by chance. A significance test was performed with a significance level of α = 0.05, which demonstrated differences between the individual groups of tested specimens. Hypothesis H0 was rejected because the tested criterion was greater than the critical value (270.21 > 2.25). The type of joint has a statistically significant effect on the measured Mmax. The mean values of the core sets from which the analyzed selections were taken differ (Table 4). The results of mechanical testing showed differences between different types of joints. The statistical analysis is presented in one-way Anova (Table 7) and two-way Anova (Table 8).

A two-way analysis of variance (ANOVA) (Table 8) was conducted to evaluate the effects of the factors Sample and Columns on the measured variable. The results indicate that both main effects, as well as their interaction, are statistically significant. The effect of Sample was found to be significant (F(6,126) = 188.88, *p* < 0.001), indicating that the different samples had a measurable impact on the response variable. Similarly, the factor Columns also showed a significant effect (F(1,126) = 357.47, *p* < 0.001), suggesting that variations across column groups influenced the outcome. Importantly, the interaction between Sample and Columns was also statistically significant (F(6,126) = 84.08, *p* < 0.001), which implies that the influence of one factor depends on the level of the other. In other words, the response variable is not only affected by each factor independently but also by their combined effect.

According to the one-way ANOVA (Table 7) and pairwise comparison conducted with the Tukey HSD test, a significant difference of α = 0.05 and a level of confidence of 95% were found between joints (Table 7). The joints were divided into four groups in the Tukey HSD test (Table 9), with two in two groups and only one in the remaining three groups.

**Figure 12:**
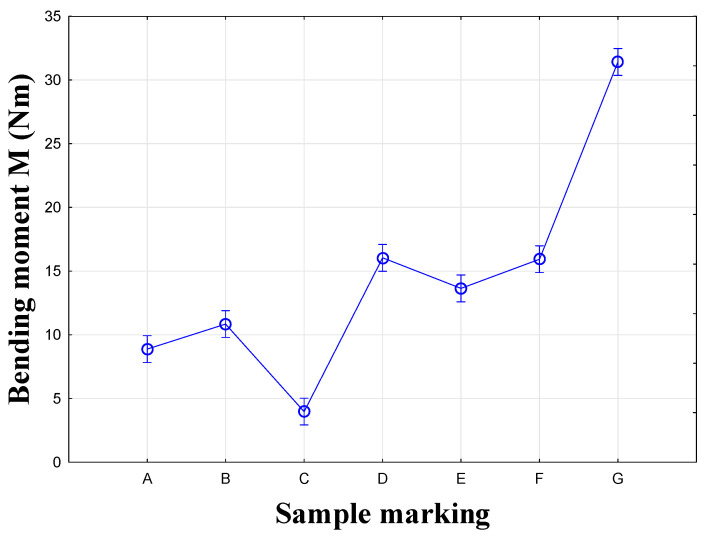
Graph for the tension test.

The first group consists of plastic biscuit joints A and B, between which a difference of 22% was found in testing. The second group consists of eccentric CAM joints D and F, with a difference of only 0.6%. The third, fourth, and fifth groups each contained one sample, C and G, which did not resemble any other joint in their testing results. The confidence intervals are depicted graphically (Figure 12). The results show that the two basic groups of specimens were not mixed: RTA joints (specimens A, B and C) and eccentric CAM joints (specimens D, E, F and G).

### 3.2. Discussion

Mechanical damage occurred on the face and rear member during testing. These deformations varied in each specimen.

**Figure 13:**
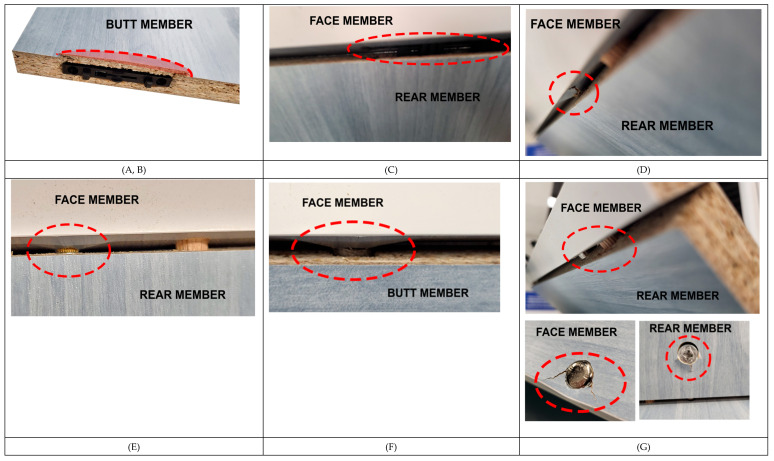
Deformation of specimens A–G.

During angular bending tests, the compressive stress was similar in specimen A (Lamello© Clamex P-14) and specimen B (Lamello© Clamex P-14 + Bisco P-15). The specimen was damaged on both the face and rear member. The joint had a tendency to tear out the remaining part of the sheet material. On the rear member, the plastic joint tore through the sheet material towards the inside of the L-shaped specimen; this phenomenon is visible in the photograph (Figure 13, specimens A and B), where this detail is highlighted with a red area. The results of the mechanical tests, where the tested joint A achieved average values of 9 Nm (tension), 15 Nm (compression), a stiffness of 55 Nm/rad (tension), and 237 Nm/rad (compression) in the bending tests in the angular plane, also confirm this. The only difference between specimen B and specimen A was an additional plastic biscuit (Lamello © Bisco P-15) in the center of the specimen, where specimen B achieved average results in the bending tests in the angular plane of 11 Nm (tension), 17 Nm (compression), a stiffness of 62 Nm/rad (tensile), and 208 Nm/rad (compression).

In comparison to research conducted by Karaman (2021) [3], who tested an L-shaped joint, namely a Clamex P-14 joint, in MDF, the current study achieved a 94% higher bending moment capacity under compression. This difference is due to the different sizes of the material used for the L-shaped joint and the absence of an edge. The cracking of the specimens is clearly visible in the photographs presented in their research. Silvana et al. (2019) [4] tested an L-shaped joint with one Clamex P-14 connector, and they achieved a 139% lower value for bending moment capacity under compression than the current study. This difference is due to the different number of connectors used and the absence of an edge.

No mechanical damage occurred in the sheet material during the testing of specimen C (Lamello© Tenso P-14), but the components were separated from each other under the load. This created a situation where the force applied to separate the components was smaller than that which is required to damage the L-shaped specimen. The photograph shows the moment just before the components were disconnected (Figure 13, specimen C), and this detail is highlighted with a red outline. According to the results of the mechanical tests, namely the bending moment capacity under compression and the bending moment capacity under tension, the joints achieved average values of 4 Nm (tension), 5 Nm (compression), a stiffness of 15 Nm/rad (tension), and 74 Nm/rad (compression). These specimens achieved the worst test results. This was also impacted by the fact that adhesive was not used in accordance with the manufacturer’s recommendation for the reasons described in Section 2.1. Sample Preparation.

Karaman (2020) [2] tested a Tenso P-14 L-shaped connector in MDF with different adhesive types and achieved a similar force value to the current research.

In the testing of specimen D (eccentric joint with metal bolt, Euro screw, and beech dowel, produced by German company), the main damage occurred on the face member, where the bolt was installed with the Euro screw. During the mechanical testing of the L-shaped joint, the screw had a tendency to fall out of the rear member, damaging the sheet material around the Euro screw. The top layer of the chipboard cracked, but the Euro screw remained firmly connected to the sheet material after the test with the Euro screw. The photograph shows the moment after the mechanical test (Figure 13, specimen D), where this detail is highlighted with a red outline. The results of the mechanical tests of joint D, namely the bending moment capacity under compression and the bending moment capacity under tension, achieved average values of 16 Nm (tension), 19 Nm (compression), a stiffness of 86 Nm/rad (tension), and 356 Nm/rad (compression).

When compared to research conducted by Zhang et al. (2024) [10], who tested an eccentric joint with a metal bolt produced in Asia on a small L-shaped joint on bamboo-oriented strand board, they achieved a 13% better bending moment capacity under compression than in our current study. Their L-shape had a length of 100 and two eccentric joints. Janikova et al. (2024) [13] tested an eccentric Minifix connector with a metal bolt added and a pre-glued dowel. They achieved a 77% better bending moment capacity under compression than in the current study and a 27% better stiffness than the current study. Their L-shaped joiner had a length of 366 mm, and they used three pre-glued dowels. Jivkov et al. (2021) [22] tested an eccentric Minifix connector with a metal bolt in lightweight panels, and due to the L-shaped joiner, they achieved a 208% lower bending moment capacity under compression than in the current study. They also achieved a 733% lower stiffness than in the current study. This was fundamentally influenced by the material used for the L-shaped joiners, their size, and the number of joints. In comparison to research conducted by Silvana (2019) [4], who tested an eccentric Minifix joint with a metal bolt on a smaller L-shaped joiner without dowels, the current study demonstrated a 58% higher bending moment capacity under compression. This difference is due to the different sizes of the L-shaped joiner and the use of dowels. Kureli and Altinok (2011) [11] tested the bending moment capacity of an L-shaped joiner with an eccentric Minifix joint. Their specimens had lengths of 200 mm, and they achieved a 43% better value than in the current study. Šimek and Konas (2009) [12] tested an eccentric Minifix joint on smaller specimens, with a length of 100 mm and a dowel without adhesive. The current study achieved a 72% higher bending moment capacity under compression. The current study’s L-shaped joiner also achieved a 111% higher value than the smaller L-shaped joiner tested by Jivkov (2002) [22]. He tested an eccentric Minifix joint without dowels. He used an ABS edge on a 100 mm L-shaped joiner.

During the testing of specimen E (an eccentric joint with a metal bolt, M4 + insert nut, and beech dowel, produced by a German company), damage mostly occurred in the rear member in which the insert nut was installed. During the mechanical testing of the L-shaped joint, the insert nut had a tendency to fall out of the rear member, and there was only minimal damage to the material around the drilled hole. This is depicted in the photograph (Figure 13, specimen E), where this detail is highlighted with a red outline. While producing specimens, it was necessary to ensure that the dowel did not rotate in the material. This would negatively affect the connection between the bolt and the dowel and the strength of the joint. The results of the mechanical tests of joint E, namely the bending moment capacity under compression and the bending moment capacity under tension, achieved average values of 14 Nm (tension), 15 Nm (compression), a stiffness of 59 Nm/rad (tension), and 233 Nm/rad (compression).

The present study achieved 146% higher values than the research specimen used in a study by Hu et al. (2024) [19]. This difference may be due to the different dimensions of the L-shaped joiner and the material used for the joining element. Guo et al. (2019) [16] tested an eccentric joint with a bolt combined with an insert nut, and they achieved a 14% higher bending moment capacity under compression than in the current study. Better results than the present study were also achieved by Kucuktuvek et al. (2017) [17]. They achieved a 75% higher bending moment capacity under compression than in the current study, but they had three eccentric joints and two wooden dowels in the specimen, and the length of the L-shaped joiner was 270 mm.

During the testing of specimen F (an eccentric joint with a metal bolt, a plastic part, a Euro screw, and a beech dowel produced by a German company), the main damage occurred on the face member where the bolt was installed. During the mechanical testing of the L-shaped joint, the euro screw had a tendency to fall out of the face member, damaging the sheet material around the euro screw. The top layer of the chipboard cracked, but the bolt remained firmly connected to the sheet material after the test with a Euro screw. The photograph shows the moment after the mechanical test (Figure 13, specimen F), where this detail is highlighted with a red outline. The results of the mechanical tests of joint F, namely the bending moment capacity under compression and the bending moment capacity under tension, achieved average values of 16 Nm (tension), 18 Nm (compression), a stiffness of 58 Nm/rad (tension), and 406 Nm/rad (compression).

Kryzaniak and Smardzewski (2020) [14] tested eccentric joints with metal bolts, plastic parts, and Euro screws on an L-shaped joiner. Their research achieved a 74% higher force value than the current research for bending moment capacity under compression. The eccentric joint and bolt used in their study were supplied by the company Hettich. Klos et al. (2018) [27] focused on testing an L-shaped eccentric joint in a small specimen. Their research achieved a 210% lower force value compared to the current research in terms of the bending moment capacity under compression. This significant difference was fundamentally influenced by the material, the size of the L-shaped specimens, and the number of joints used. Smardzewski et al. (2014) [15] obtained results similar to those of the current study. Their research involved testing L-shaped joints with an eccentric joint consisting of a metal bolt, a plastic component, a Euro screw, and a wooden dowel. The dimensions of their specimens were comparable to those used in the present study.

The main damage in the testing of specimen G (an eccentric joint with a metal-capped bolt, Euro screw, and beech dowel, produced by a German company) occurred in both the face and rear members. The mechanical testing of joint L resulted in the deformation of the material around the bolt head on the longitudinal surface. The photograph shows the moment after the mechanical test (Figure 13, specimen G), where this detail is highlighted with a red outline. The bolt head tended to press into the material, causing damage to the area around it. The eccentric joint was also pushed into the rear member, and this resulted in damage to the area around it. The capped bolt itself was also bent. The results of the mechanical tests of joint G, namely the bending moment capacity under compression and the bending moment capacity under tension, achieved average values of 31 Nm (tension), 47 Nm (compression), a stiffness of 91 Nm/rad (tension), and 242 Nm/rad (compression).

Similar values for the bending moment capacity under compression were also achieved in research by Kasal et al. (2008) [28] when testing L-shaped joiners, which they connected using three screws with dimensions of 4×50 mm. They achieved a bending moment of 45 Nm, which is approximately 4% less than that achieved in the current study. Therefore, it can be concluded that using three screws (4×50 mm) for the connection, or an eccentric joint with a metal-capped bolt, Euro screw, and dowel, achieves similar results. An L-shaped joiner connected by screws (4 × 50 mm) was tested by Kasal et al. (2010), where the specimen was also adhesive [29]. They proved that adding adhesive would increase the strength even more.

The inverse relationship between the cost of the coupling and the results of peak-load testing has already been confirmed in a previous study. Therefore, more expensive joints cannot be expected to perform better during bending in the angular plane. As such, the results of the maximum load tests must be taken into account when evaluating joint performance.

This study showed that the joint that connected the two parts of the specimen through the materials themselves achieved the best performance among all the tested specimens. This finding is consistent with those reported in other studies [4,9,15,22].

Future research could build upon the findings of this study in several ways. First, testing could be extended to include a wider variety of joint types and material combinations in order to assess whether the observed trends hold under different structural conditions, for example, plywood and solid wood in MDF. It would also be beneficial to evaluate joint performance under more realistic loading scenarios, such as cyclic or environmental stress, to simulate real-life use. Additionally, further investigation into the cost–performance ratio of the joints could provide valuable insights for practical applications, especially in construction or furniture design, where both mechanical performance and cost efficiency are critical. Finally, the experimental results presented in this study may serve as a reference for validating numerical simulations using finite element modeling, enabling more advanced analysis of joint behavior under various stress conditions.

## 4. Conclusions

The results of the mechanical testing of L-shaped specimens show that there are differences between the tested joints. When an eccentric joint is used with just a metal bolt or an eccentric joint is used with a metal bolt with a plastic part and the addition of a wooden dowel, similar results are achieved, so it can be concluded that there is no noticeable difference between them. An eccentric joint with a metal-capped bolt achieved the best results in all the tests. This joint will achieve 147% higher values for the bending moment capacity under compression than a standard eccentric joint with a Euro screw bolt, and it will achieve 213% higher values for the bending moment capacity under compression than Lamello© and Clamex P-14 joints.

The Lamello© joints did not support the idea that higher-price joints achieve higher strength than eccentric joints. The Clamex P-14 joints achieved a similarly or worse result than the eccentric joints. If Lamello© Bisco P-15 joints are added to the plastic Clamex P-14 joint, this joint will achieve 13% higher values for the bending moment capacity under compression and 22% higher values for the bending moment capacity under tension. The plastic Tenso P-14 joint should be combined with adhesive.

This study underscores the critical importance of comprehensively understanding various furniture joint systems. The results obtained not only reveal significant differences in efficiency and strength among the tested connectors but also illustrate their potential influence on the speed, flexibility, and overall quality of furniture assembly. The significance of this work lies in its provision of valuable empirical data that may assist manufacturers, designers, and engineers in selecting optimal joining technologies tailored to specific functional and aesthetic requirements. Moreover, these findings establish a foundation for future research focused on the optimization of joint designs, the development of innovative fastening solutions, and the advancement of production processes aimed at enhancing durability, sustainability, and user-centric functionality within furniture construction.

This research did not receive any specific grant from funding agencies in the public, commercial, or not-for-profit sectors.

## Figures and Tables

**Table 2:** Average bending moment capacity under compression.

Specimen Type	N	Mean (Nm)	Stiffness (Nm/rad)	Variance (%)
A	10	15	237	8.78
B	10	17	208	1.44
C	10	5	74	0.12
D	10	19	356	4.53
E	10	15	233	5.02
F	9	18	406	2.58
G	10	47	242	3.84

**Table 3:** Average bending moment capacity under tension.

Specimen Type	N	Mean (Nm)	Stiffness (Nm/rad)	Variance (%)
A	10	9	55	4.19
B	10	11	62	2.31
C	10	4	15	0.15
D	10	16	86	5.57
E	10	14	59	1.75
F	10	16	58	2.51
G	10	31	91	2.82

**Table 4:** Summary of the one-way ANOVA results for the compression test.

Source of Variance	Sum of Squares	Degrees of Freedom	Mean Squares	F-Value	*p*-Value	F-Critical
Between groups	10,067.12	6	1677.858	444.23	5.74	2.25
Within groups	234.17	62	3.78			
Total	10,301.3	68				

**Table 5:** Summary of the two-way ANOVA results for the compression test.

Source of Variance	Sum of Squares	Degrees of Freedom	Mean Squares	F-Value	*p*-Value	F-Critical
Sample	8794.79	6	1465.79	352.97	3.02 × 10^−76^	2.17
Columns	604.54	1	604.54	145.57	9.37 × 10^−23^	3.91
Interaction	2415.42	6	402.57	96.94	8.63 × 10^−45^	2.17
Within	523.25	126	4.15			
Total	12,338.01	139				

**Table 6:** Tukey HSD analysis of the differences between the homogeneity groups with a confidence interval of 95% for the compression test.

Specimen Type	Mean (M/Nm)	Homogeneity Groups			
		1	2	3	4
C	5			***	
A	15	***			
E	15	***			
B	17	***	***		
F	18		***		
D	19		***		
G	47				***

*** indicating membership of the group.

**Table 7:** Summary of the one-way ANOVA results for the tension test.

Source of Variance	Sum of Squares	Degrees of Freedom	Mean Squares	F-Value	*p*-Value	F-Critical
Between groups	4469.03	6	744.84	270.21	5.64	2.25
Within groups	173.66	63	2.76			
Total	4642.69	69				

**Table 8:** Summary of the two-way ANOVA results for the tension test.

Source of Variance	Sum of Squares	Degrees of Freedom	Mean Squares	F-Value	*p*-Value	F-Critical
Sample	3302.88	6	550.48	188.88	1.75 × 10^−60^	2.17
Columns	1041.82	1	1041.82	357.47	1.33 × 10^−38^	3.91
Interaction	1470.23	6	245.04	84.078	1.18 × 10^−41^	2.17
Within	367.21	126	2.91			
Total	6182.15	139				

**Table 9:** Tukey HSD analysis of the differences between the homogeneity groups with a confidence interval of 95% for the tension test.

Specimen Type	Mean (M/Nm)	Homogeneity Groups				
		1	2	3	4	5
C	4			***		
A	9	***				
B	11	***				
E	14				***	
F	16		***			
D	16		***			
G	31					***

*** indicating membership of the group.

## Data Availability

Dataset available on request from the authors.
